# Investigation into Thermal Decomposition Mechanisms of Al-Coated CL-20-Based Cocrystal Explosives via Reactive Molecular Dynamics Simulations

**DOI:** 10.3390/ijms272110047

**Published:** 2026-11-09

**Authors:** Yuhao Shi, Xinyu Li, Jincheng Ji

**Affiliations:** Key Laboratory of Drug Synthesis and Optimization, Jingchu University of Technology, Jingmen 448000, China; 18371115667@163.com (X.L.); forbiddenciy_jjc@163.com (J.J.)

**Keywords:** Al-coated structure, CL-20-based cocrystal, thermal decomposition, diffusion analysis, molecular dynamics

## Abstract

This study used reactive molecular dynamics (RMD) simulations to systematically investigate the thermal decomposition characteristics of Al-coated CL-20-based cocrystal explosive (CL-20/BTF@Al and CL-20/TNT@Al) nanoparticles at 2500 K, revealing how metallic Al exerts regulatory effects on the thermal decomposition mechanisms of energetic materials compared with the pure CL-20-based cocrystal system. The results indicate that the Al-coated structure could create a large number of local high-temperature hotspots in the system, providing active sites to initiate reactions, while slowing down heat loss and increasing the system temperature. The evolution of thermal decomposition products indicates significant differences between the Al-coated systems and the pure cocrystal systems, confirming that Al effectively regulates the thermal decomposition pathway. Atomic diffusion analysis shows that the exothermic reaction of Al promotes the decomposition of intermediates, increases the content of small molecules containing free H and N, and thereby enhances the diffusion coefficients of H and N atoms. In addition, Al combines with O to form stable clusters, which adsorb bound carbon fragments at their interfaces, significantly inhibiting the migration and diffusion of C and O atoms, thereby reducing their diffusion coefficients. This work clarifies the microscopic thermal reaction mechanism of metal/energetic composites from an atomic-scale perspective, and lays an essential theoretical foundation for component formulation and performance optimization of high-efficiency energetic composites.

## 1. Introduction

2,4,6,8,10,12-Hexanitro-2,4,6,8,10,12-hexaazaisowurtzitane (CL-20) has a cage structure containing six -NO_2_ groups, and its density and heat of formation are higher than those of monocyclic nitramine explosives, so it can release a large amount of energy during the decomposition process [1,2,3]. It is currently known as the highest-energy non-nuclear elemental explosive that can be practically applied, but its high sensitivity limits its further application. Cocrystallization technology is an effective material modification method that can combine two or more components through intermolecular interactions such as hydrogen bonds, π-stacking, or van der Waals forces without damaging the original molecular structure of the explosives, thereby modulating explosive performance and sensitivity [4,5,6]. Due to the promising application prospects of CL-20, researchers have modified its sensitivity through cocrystallization technology, thereby enhancing its overall performance. Bolton et al. prepared a CL-20/2,4,6-trinitrotoluene (TNT) cocrystal explosive in 2011 and analyzed and characterized its structure and properties. The results indicate that the molar ratio of the two molecules in the unit cell is 1:1, the driving forces for cocrystal formation are intermolecular C-H∙∙∙O hydrogen bonds, and the impact stability of cocrystal was significantly improved compared to pure components [7]. Yang et al. prepared CL-20/BTF cocrystal at a molecular molar ratio of 1:1 in 2012. The test results showed that C-H∙∙∙N hydrogen bonds and π-π interactions were the main driving forces for the formation of CL-20/benzotrifuroxan (BTF) cocrystal, and the cocrystal had much higher detonation performance than the original crystal [8]. Bennion et al. synthesized an energetic cocrystal with a molar ratio of 2:1 between CL-20 and H_2_O_2_ by introducing H_2_O_2_ into the CL-20 unit cell. The test results showed that the detonation velocity and pressure of the CL-20/H_2_O_2_ cocrystal are greatly improved compared to the pure component CL-20 crystal [9]. Ma et al. prepared a CL-20/1-methyl-3,4,5-trinitropyrazole (MTNP) cocrystal explosive with a molar ratio of 1:1. The results showed that the CL-20/MTNP cocrystal had high energy density (1.93 g·cm^−3^) and excellent detonation performance, with a detonation velocity and pressure of 9347 m·s^−1^ and 40.5 GPa, respectively [10]. These research results indicate that cocrystal technology has significant advantages in improving the sensitivity and enhancing the comprehensive properties of CL-20.

The reaction of explosives is essentially a process of rapid release of energy. Nano metal powders are usually added to explosives to enhance the energy released and reaction stability in applications, thereby significantly increasing the power of explosives [11,12,13]. Aluminum (Al) powder is widely used in solid propellants and mixed explosives due to its excellent combustion performance, which makes the comprehensive performance of Al-containing explosives significantly better than that of pure organic crystal explosives [11,14]. The reaction process of explosives involves complex physical and chemical coupling mechanisms, accompanied by extremely high-temperature and high-pressure environments. The multiphase interactions within the system make the reaction mechanism more complex and intricate after introducing Al powder; therefore, studying the reaction process through experimental methods is challenging. Molecular dynamics (MD) simulations can overcome experimental limitations by probing the reaction evolution process from a microscopic perspective, and they have been widely used in the study of the decomposition and combustion mechanisms of energetic materials [15,16,17]. Reactive molecular dynamics (RMD) simulations have successfully revealed the thermal decomposition and combustion mechanisms of a series of Al-containing explosive systems, such as 1,3,5-trinitro-1,3,5-triazinane (RDX) with the Al_2_O_3_ system [18], AlH_3_ nanoparticles (NPs) with the 2,2′,4,4′,6,6′-hexanitrodiphenyl ethylene (HNS) system [19], Al NPs with the 2,4,6-trinitro-1,3,5-triaminobenzene (TATB) system [20], and so on. These studies indicate that RMD simulation can accurately describe the thermal decomposition process of Al-containing explosives.

Due to the more uniform distribution of metals in explosive/metal nanocomposite systems with core–shell structures, these systems are more conducive to improving the thermal reactivity of explosives [21,22]. The relevant experimental studies are as follows: Jin et al. introduced Al and CuO NPs onto the 1,3,5,7-tetranitro-1,3,5,7-tetraazacyclooctane (HMX) surface through biological self-assembly to prepare the HMX@Al@CuO nanoenergetic composite, and the thermal analysis results showed that compared to the physically mixed sample, the HMX@Al@CuO system not only increased the total heat of the reaction, but also reduced the activation energy of HMX by 41% [23]. Guo et al. prepared a core–shell-structured CL-20/TNT@Al composite, and the results showed that the cocrystal size of CL-20/TNT was about 20–40 μm, and the nano-Al powder is uniformly coated on the cocrystal [24]. In terms of theoretical research, Sun et al. used RMD simulations to study the thermal decomposition process of core–shell-structured RDX@AlH_3_, HMX@AlH_3_ and CL-20@AlH_3_ NPs. The results showed that the decomposition of CL-20@AlH3 occurred earlier, and the introduction of the AlH_3_ shell did not change the initial decomposition mechanism of RDX, CL-20, and HMX. The Al-containing clusters generated during the decomposition process are the main components of the solid product [25]. Ji et al. studied the combustion process of CL-20@Al and HMX@Al in an oxygen atmosphere via RMD simulations. It was found that the Al shell underwent a melting diffusion reaction aggregation process from the initial surface shell to the bulk, and O_2_ rarely directly participated in the initial decomposition of explosive molecules, mainly reacting with intermediates and free radicals. In addition, the reaction rate of CL-20 was higher than that of HMX [26].

Therefore, Al-coated CL-20-based cocrystal NPs with a core–shell structure (CL-20/BTF@Al and CL-20/TNT@Al) were constructed in this work, and RMD simulations were used to study the thermal decomposition mechanisms of the Al-containing cocrystal explosives at high temperatures of 2500 K. The thermal decomposition process of these Al-coated CL-20-based cocrystal NPs was compared with that of pure CL-20/BTF and CL-20/TNT NPs, and the influence of Al on the thermal decomposition mechanism of CL-20-based cocrystal explosives was investigated. This study can offer theoretical support for revealing the thermal reaction mechanism of energetic composite materials, and further expand the research scope of cocrystal explosives/Al composites.

## 2. Results and Discussion

### 2.1. Evolution of Hotspots

**Figure 1:**
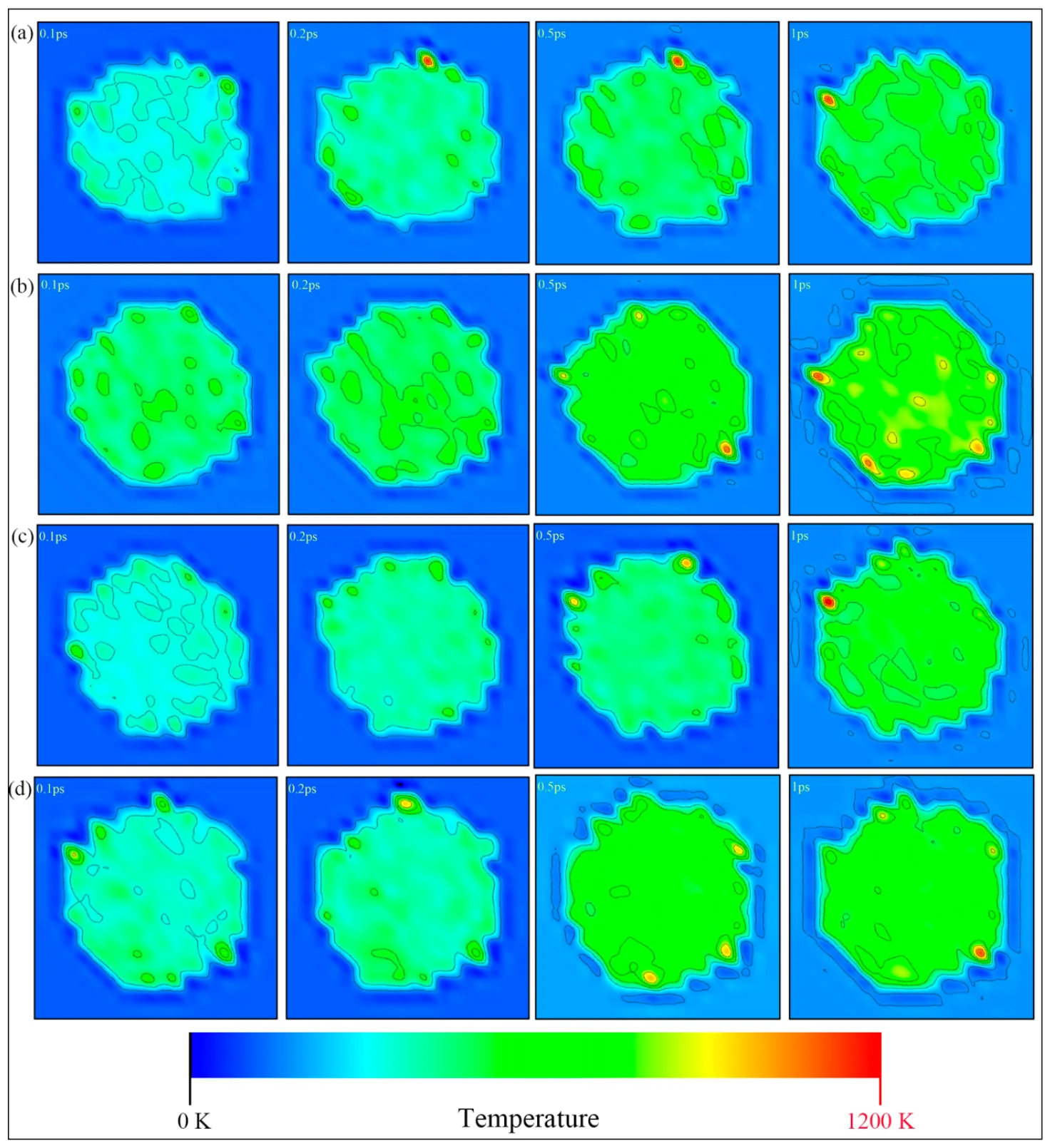
The local temperature distribution of different systems: CL-20/BTF (**a**), CL-20/BTF@Al (**b**), CL-20/TNT (**c**), and CL-20/TNT@Al (**d**).

In order to understand the formation and evolution process of high-temperature regions in Al-coated CL-20-based cocrystal explosives, the system was partitioned uniformly into multiple equal-sized regions according to the equal energy distribution principle. Afterwards, the average atomic temperature within each region was computed, and a two-dimensional temperature-field contour map was produced [27,28]. Figure 1 shows the temperature distribution of different systems during the initial stage of the reaction. The overall average temperature of the system was far below 2500 K in the early stages. The system produced local hotspots due to the surface effect of the NPs and the influence of the Al shell, and the hotspot temperature was higher than the average temperature of the system at that time, but still significantly lower than 2500 K. The surface temperature of CL-20/BTF NPs first increased, followed by an overall temperature increase in the system (the temperature range gradually changes from light green to dark green), and finally a hot spot formed on the surface of the nanoparticles (the red area), as shown in Figure 1a. This is because molecules on the surface of explosive NPs have much higher reactivity, making them more prone to decomposition reactions and heat release. Therefore, the entire process started from the surface and gradually advanced towards the interior of the particles. It can be seen from Figure 1b that the temperature evolution trend of CL-20/BTF@Al NPs was consistent with that of CL-20/BTF NPs as a whole, following the process of surface heating, overall heating, and hot spot formation. Although local hotspots only formed at 0.5 ps, the overall temperature of the system was higher at the same time. The temperature variation trend of CL-20/TNT and CL-20/TNT@Al NPs in the early stage of the reaction system also followed a similar pattern. This is because the presence of the Al shell slowed down the rate of heat loss in the system to a certain extent, thereby promoting an increase in the temperature of the system. The Al-coated structure was able to generate abundant localized high-temperature hotspots inside the system. These hotspots triggered fast chemical reactions and maintained ongoing reaction evolution, thus acting as the core nucleation zones for reaction initiation [29].

### 2.2. Morphology Evolution

**Figure 2:**
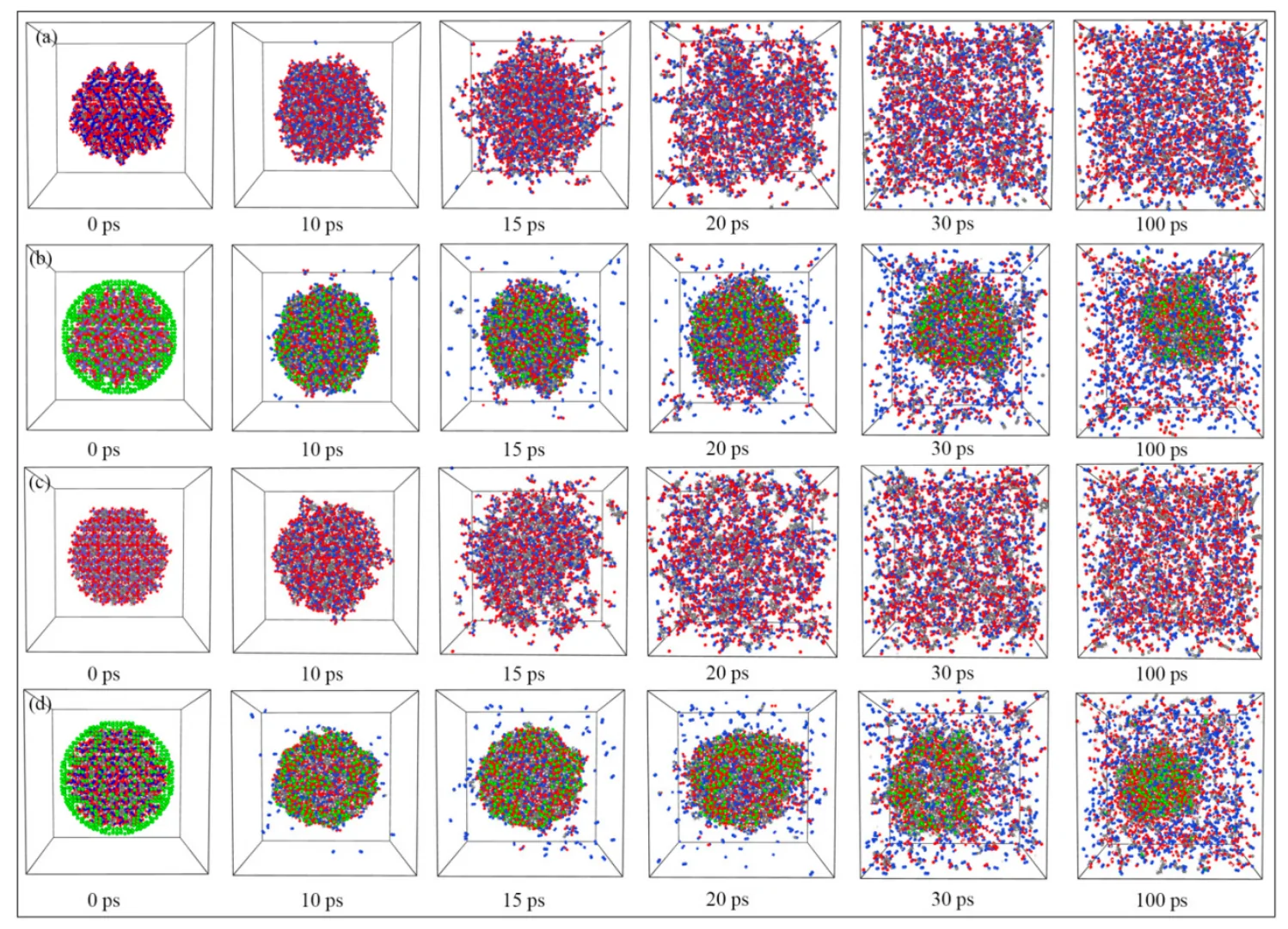
The snapshots of thermal decomposition process: CL-20/BTF (**a**), CL-20/BTF@Al (**b**), CL-20/TNT (**c**), and CL-20/TNT@Al (**d**). The C, H, O, N and Al are represented by gray, white, red, blue and green balls, respectively.

In order to gain a comprehensive understanding of the thermal decomposition process of Al-coated CL-20-based cocrystal explosives at high temperatures, the changes in the system morphology at different time points were studied, as presented in Figure 2. Figure 2a shows that CL-20/BTF NPs will first undergo expansion during the thermal decomposition process. The expansion degree of CL-20/BTF NPs is relatively low in the range of 0–10 ps, and more surface molecules became disordered due to the overall low temperature of the system during this time period. CL-20/BTF NPs underwent significant expansion and began to decompose rapidly at 15 ps, and a noticeable number of gas molecules were generated. As the reaction proceeded, decomposition continued. The NPs had completely decomposed by 100 ps, and the system was populated with decomposition intermediates and free radicals. Volume expansion also occurred for the CL-20/BTF@Al NPs during decomposition, as shown in Figure 2b, but the degree of expansion is smaller than that of CL-20/BTF NPs at the same time. This is because the Al shell forms a physical barrier to the diffusion of substances, thereby suppressing particle expansion. There were obvious gas molecules escaping from the Al shell at 10 ps, indicating that CL-20 and BTF molecules had undergone significant decomposition reactions. The system was filled with large amounts of decomposition products at 30 ps. From 30 ps to the end of the simulation, the decomposition reaction continued, while a series of complex chemical reactions continued to occur between various decomposition products. Figure 2c and Figure 2d show snapshots of the thermal decomposition processes of CL-20/TNT and CL-20/TNT@Al, respectively. It can be observed that their thermal decomposition processes resemble those of CL-20/BTF and CL-20/BTF@Al NPs, and both cases feature a disordering of surface molecules, followed by NPs expansion and subsequent decomposition reactions. Comparative analysis reveals that CL-20/BTF@Al and CL-20/TNT@Al NPs underwent thermal decomposition earlier than CL-20/BTF and CL-20/TNT NPs. This phenomenon is attributed to the Al shell, which reduces the heat-loss rate of the system. This effect promotes temperature increase and keeps the explosive molecules inside the Al shell at a higher-energy state, thereby promoting the onset of decomposition reactions.

**Figure 3:**
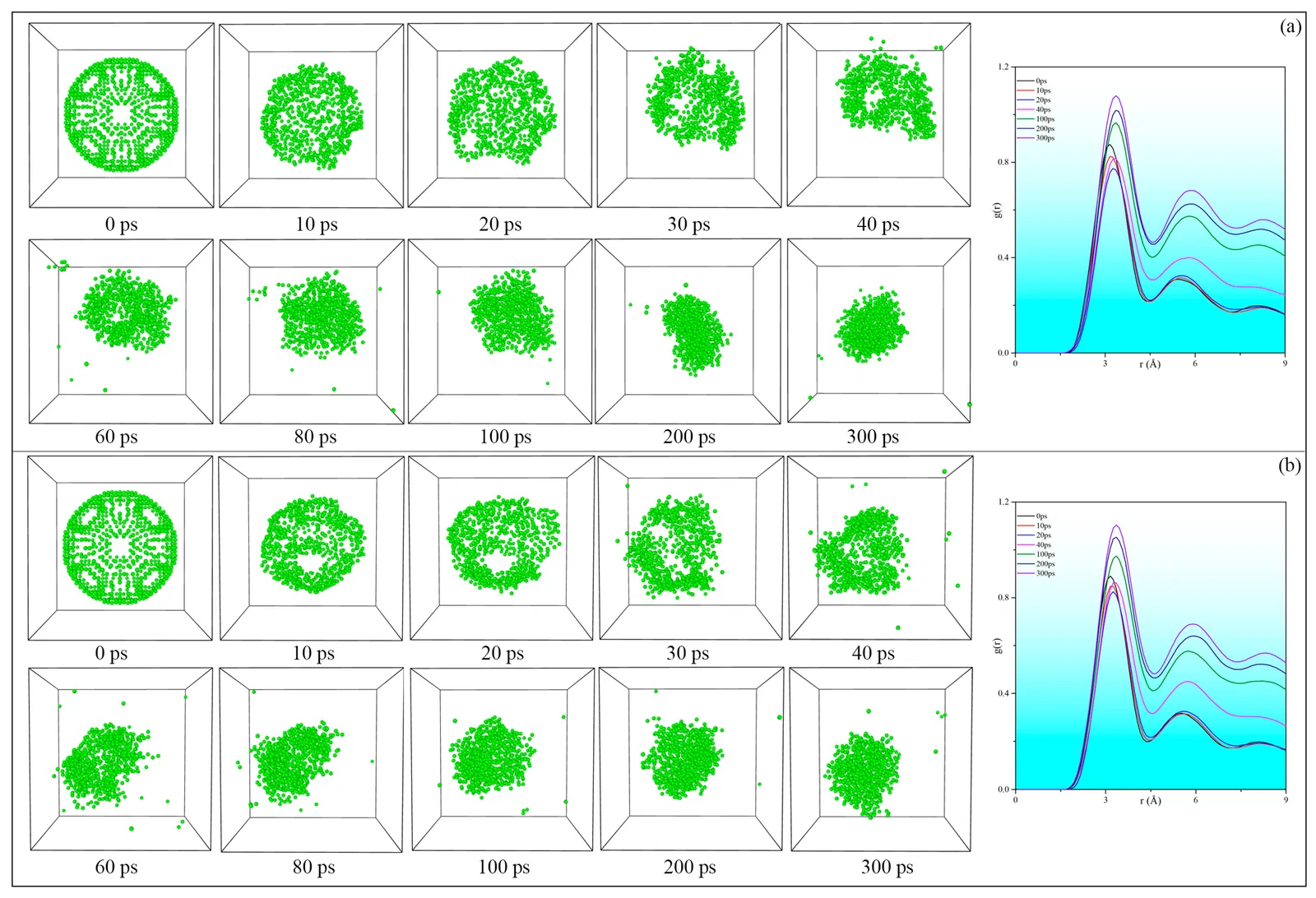
The morphology evolution of Al shell and the RDFs distribution of Al-Al: CL-20/BTF@Al (**a**) and CL-20/TNT@Al (**b**). Al is represented by green balls.

During the thermal decomposition of Al-coated CL-20-based cocrystal explosives, the Al shell experiences a series of successive reactions, gradually converting its original shell-type configuration into a bulk-like structure, as shown in Figure 3. Figure 3a illustrates the morphological evolution of the Al shell during the thermal decomposition of the CL-20/BTF@Al system. Al atoms were regularly assembled to form an intact shell configuration at 0 ps, and the ordered Al shell began to become disordered at 10 ps, while retaining its overall shell-like feature. The Al shell began to melt, the shell structure loosened with the proceeding reaction. The Al shell had fully melted into a disordered amorphous structure by 30 ps. Al atoms began to aggregate gradually at 40 ps, and they had agglomerated into block-shaped clusters containing internal voids by 60 ps. As the reaction further proceeds, these inner voids within the clusters continuously shrink and finally disappear at 200 ps, forming a denser amorphous block cluster. The transformation of the Al shell into a dense block can be more accurately described using the radial distribution function (RDF). It can be seen from Figure 3a that the intensity of the first peak of RDFs decreased gradually within 0–20 ps, and the value of r gradually shifted to the right, indicating that the interatomic interaction among Al atoms was weakened and their average interatomic distance rose, corresponding to the melting behavior of the Al shell. The first-peak intensity of RDFs increased progressively after 40 ps, indicating the onset of Al atoms aggregation and the final formation of a densified amorphous bulk cluster. The morphology evolution of the Al shell during the thermal decomposition of CL-20/TNT@Al resembles that of CL-20/BTF@Al, as illustrated in Figure 3b. In both systems, the Al shell initially melted, after which Al atoms agglomerated into loose clusters. These clusters contracted continuously as the internal voids shrank and eventually developed into denser amorphous bulk clusters.

### 2.3. Decomposition Products

**Figure 4:**
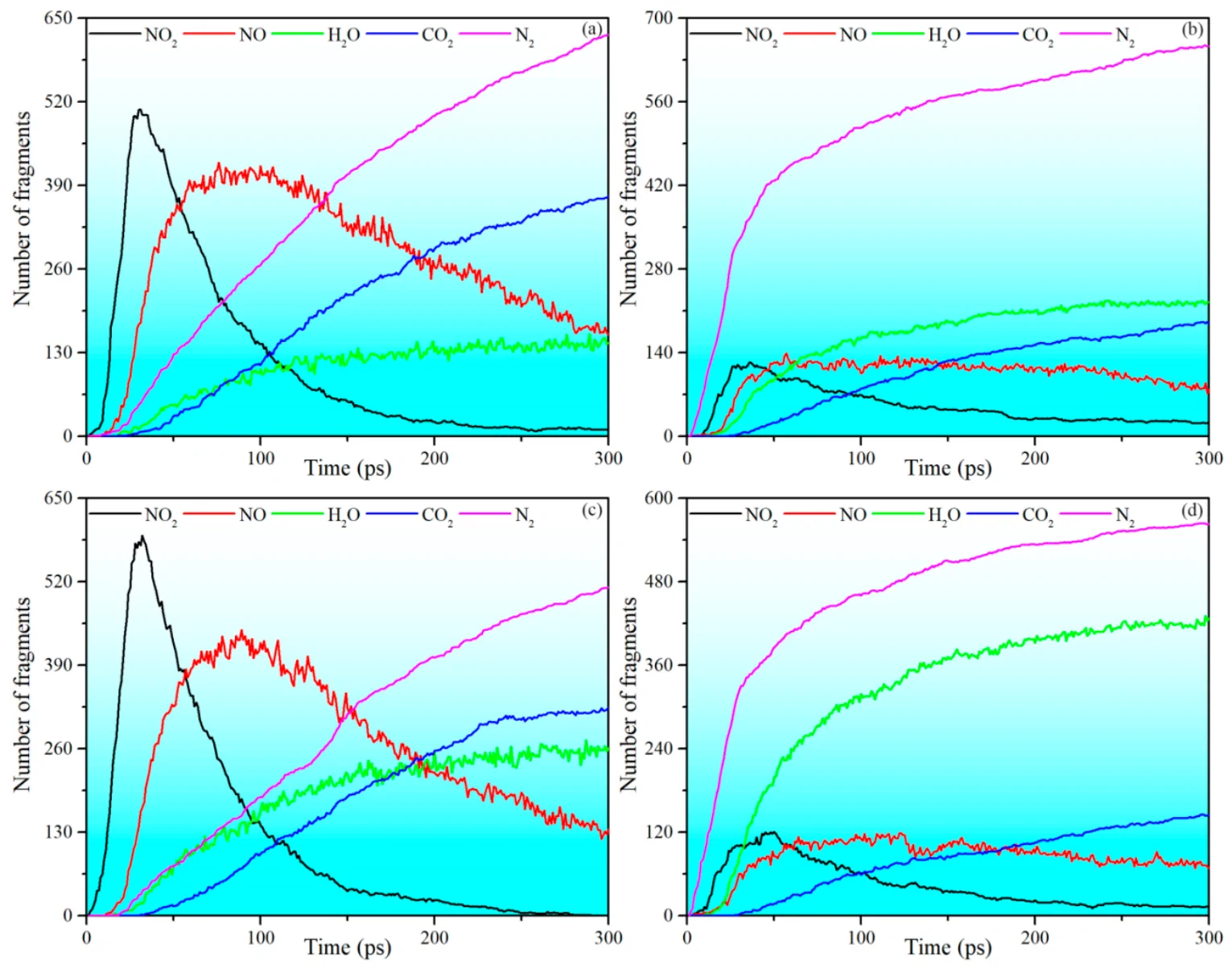
Quantity variation in products with time: CL-20/BTF (**a**), CL-20/BTF@Al (**b**), CL-20/TNT (**c**), and CL-20/TNT@Al (**d**).

Energetic materials would generate diverse products under high-temperature conditions, and the time-dependent quantity variations in intermediate products (NO and NO_2_) and final products (N_2_, H_2_O, and CO_2_) in different systems during thermal decomposition are statistically analyzed, as presented in Figure 4. It can be seen from Figure 4a that NO_2_ was the earliest accumulating product during the thermal decomposition of CL-20/BTF NP, and the amount of NO_2_ increased rapidly, reaching its maximum value at 30 ps and then rapidly decreasing. This is attributed to the high reactivity of NO_2_ at a high temperature, which enables it to act as an intermediate and take part in subsequent reactions. Therefore, the amount of NO_2_ would rapidly decrease after reaching its peak. The trend of changes in the quantity of NO is similar to that of NO_2_, both experiencing a process of first increasing and then decreasing, and the peak of NO appears later than NO_2_; its quantity is also lower than NO_2_. This is because NO_2_ is a primary decomposition product, so its quantity rapidly increases and peaks first, while NO is mainly generated through secondary reduction reactions between NO_2_ and reducing components such as C and H radicals, and belongs to the secondary gas phase product. The quantities of N_2_, H_2_O, and CO_2_ had been continuously increasing from their appearance to the end of the simulation. N_2_ has the highest quantity, followed by CO_2_, and H_2_O has the lowest quantity. The trend of this quantity change was due to the fact that N_2_, H_2_O, and CO_2_, as the final products of thermal decomposition, struggled to undergo secondary decomposition within the temperature range of thermal decomposition. Various active intermediate fragments continuously transform into three stable small molecules as the reaction proceeds, and the number of products continues to increase. The maximum amount of N_2_ was due to the fact that both CL-20 and BTF were high-nitrogen and energetic components, with abundant nitrogen reserves in the system. Various nitrogen-containing free radicals and nitrogen oxide intermediates were ultimately converted into the thermodynamically stable N_2_ in large quantities. There is obvious competition for carbon conversion, and only a portion of carbon further oxidized to obtain CO_2_. At the same time, there are incompletely oxidized carbon-containing intermediates or even solid-phase residual carbon, which limits the amount of CO_2_ generated. In addition, H radicals are the smallest and most active, and they can participate in the generation of various intermediates. Therefore, only some H atoms participate in the generation of H_2_O, resulting in the lowest amount of H_2_O generated.

N_2_ was the initial accumulating product in the thermal decomposition process of CL-20/BTF@Al NP, as illustrated in Figure 4b. However, it was found that NO_2_ was actually the primary species formed by tracking the trajectory file. The highly reactive nano-Al could swiftly reduce NO_2_ and weaken its accumulation. This greatly promotes the conversion of intermediates toward more thermodynamically stable N_2_, resulting in a large accumulation of N_2_ first. The quantity of N_2_ rose sharply from 0 to 30 ps, followed by a slow continuous increase until the simulation terminates. The amount of NO_2_ presented an upward then downward trend, and reached its maximum value at 30 ps, but its maximum value was substantially lower than that observed for the CL-20/BTF system. The amount of NO did not significantly decrease after reaching its maximum value until the end of the simulation, but remained constantly fluctuating. The amount of H_2_O rose sharply in the range of 0 to 50 ps, and then gradually grew until the simulation was completed. CO_2_ increased slowly throughout the whole process, and its content remained lower than that of H_2_O in this system. This phenomenon originated from the strong oxygen affinity of Al under high-temperature conditions, and Al preferentially captured O atoms and suppressed the full oxidation of C. Meanwhile, Al reacted with C-containing fragments to form clusters, which directly consumed part of the gaseous C-containing sources.

The variations in product quantity during the thermal decomposition of CL-20/TNT NPs are presented in Figure 4c, and the trend of product quantity variation is generally similar to that in the CL-20/BTF system, with the main difference being the complete consumption of NO_2_ by the end of simulation in the CL-20/TNT system. This is because TNT molecules contained a large amount of reducing groups such as methyl and aromatic rings, and the thermal decomposition process could continuously release a large amount of reducing free radicals. In contrast, BTF was an oxidized furazan structure, which decomposed to generate oxygen-containing free radicals, resulting in a stronger overall oxidizing property of the system. Sufficient reducing free radicals continuously undergo secondary reduction reactions with NO_2_ in the CL-20/TNT system, which drives NO_2_ to continuously be converted into other products until complete consumption. The CL-20/BTF system had a strong oxidizing atmosphere, which suppressed the reduction consumption of NO_2_, so residual NO_2_ still existed at the end of the simulation. The variation in product quantity during the CL-20/TNT@Al decomposition process in Figure 4d was consistent with that of CL-20/BTF@Al, except that the amount of H_2_O in the CL-20/TNT@Al system was significantly higher than that in the CL-20/BTF@Al system at the end of the simulation. This is because TNT molecules contained hydrogen-rich structures such as methyl groups, and thermal decomposition could release a large amount of hydrogen free radicals, providing sufficient hydrogen sources for the generation of H_2_O. Since BTF molecules contain no hydrogen, the CL-20/TNT@Al system had a higher total number of H atoms available for binding to form H_2_O than the CL-20/BTF@Al system.

**Figure 5:**
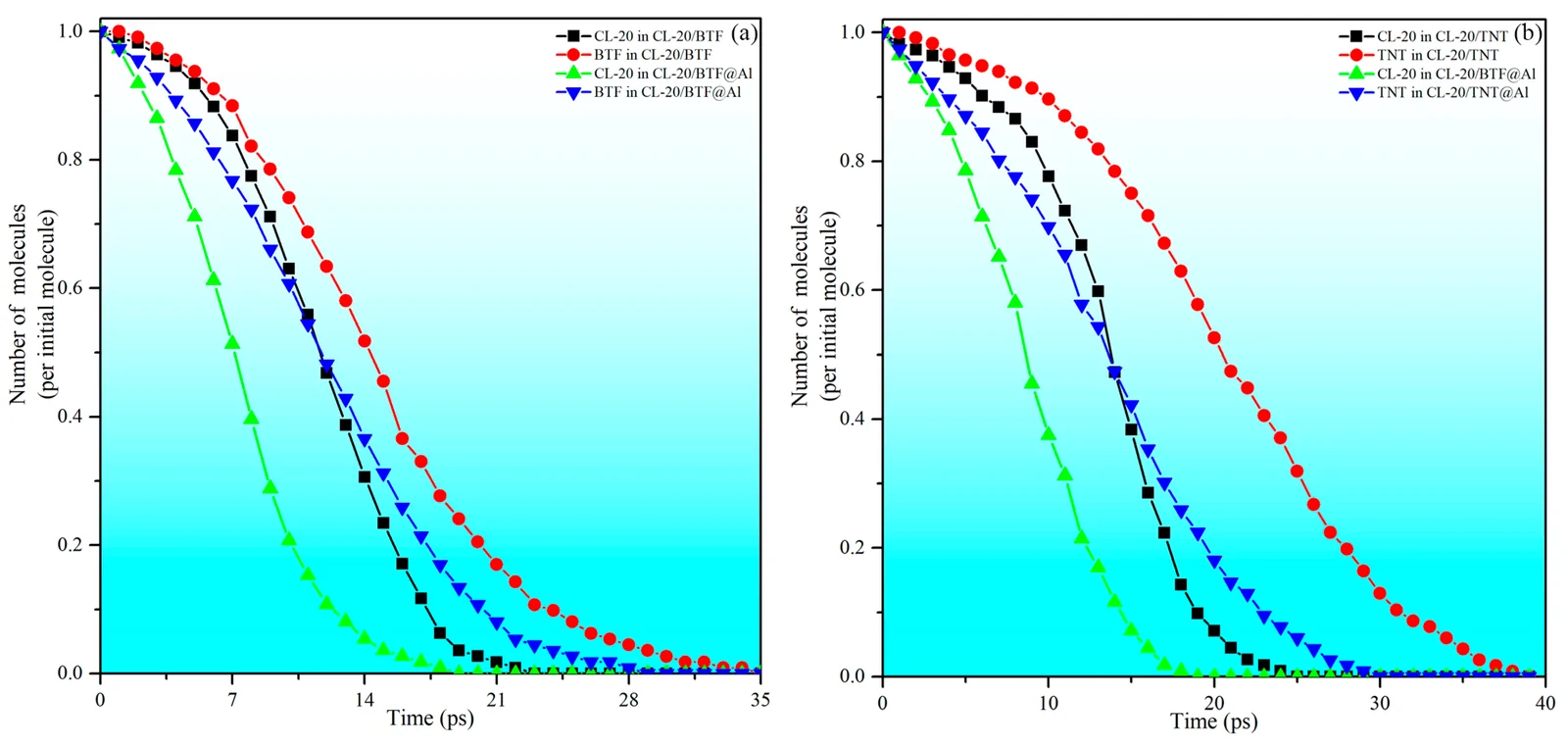
Quantity variation in explosive molecules with time during the thermal decomposition process. (**a**) CL-20/BTF and CL-20/BTF@Al, (**b**) CL-20/TNT and CL-20/TNT@Al.

It can be seen that the decomposition occurred earlier for Al-coated CL-20-based cocrystal NPs than in pure CL-20-based cocrystal NPs by examining the variations in product amounts, and this is consistent with the reaction process shown in Figure 2. Figure 5 shows the evolution trend of the number of explosive molecules with time during the thermal decomposition process. Under the same simulation conditions, CL-20 molecules underwent the initial reaction the earliest, followed by BTF molecules, and TNT molecules underwent it the latest. This is because CL-20 molecules have the lowest stability at high temperatures, and initial decomposition is most likely to occur, resulting in the earliest occurrence of initial reactions. The stability of BTF was higher than that of CL-20 but lower than that of TNT; therefore, the initial reaction occurred at an intermediate time. TNT had a stable conjugated aromatic skeleton, and the conjugation between nitro groups and benzene rings enhanced its molecular stability. The energy required for bond cleavage was the highest, and the time required to initiate decomposition was the latest. It is worth noting that whether it was the CL-20/BTF or CL-20/TNT system, the time for the complete reaction of explosive molecules after Al-coating was earlier than that in the pure cocrystal system. This was because the Al-coated layer formed a micro-confinement structure, which slowed down the diffusion of internal heat and gas-phase active free radicals, promoted local heat accumulation and increased the concentration of internal free radicals, and enhanced the self-catalytic effect of thermal decomposition. In addition, the exothermic oxidation of active Al provided an additional heat source, which jointly promoted the accelerated decomposition of explosive molecules and shortened the time required for complete reaction.

### 2.4. Diffusion Analysis

**Figure 6:**
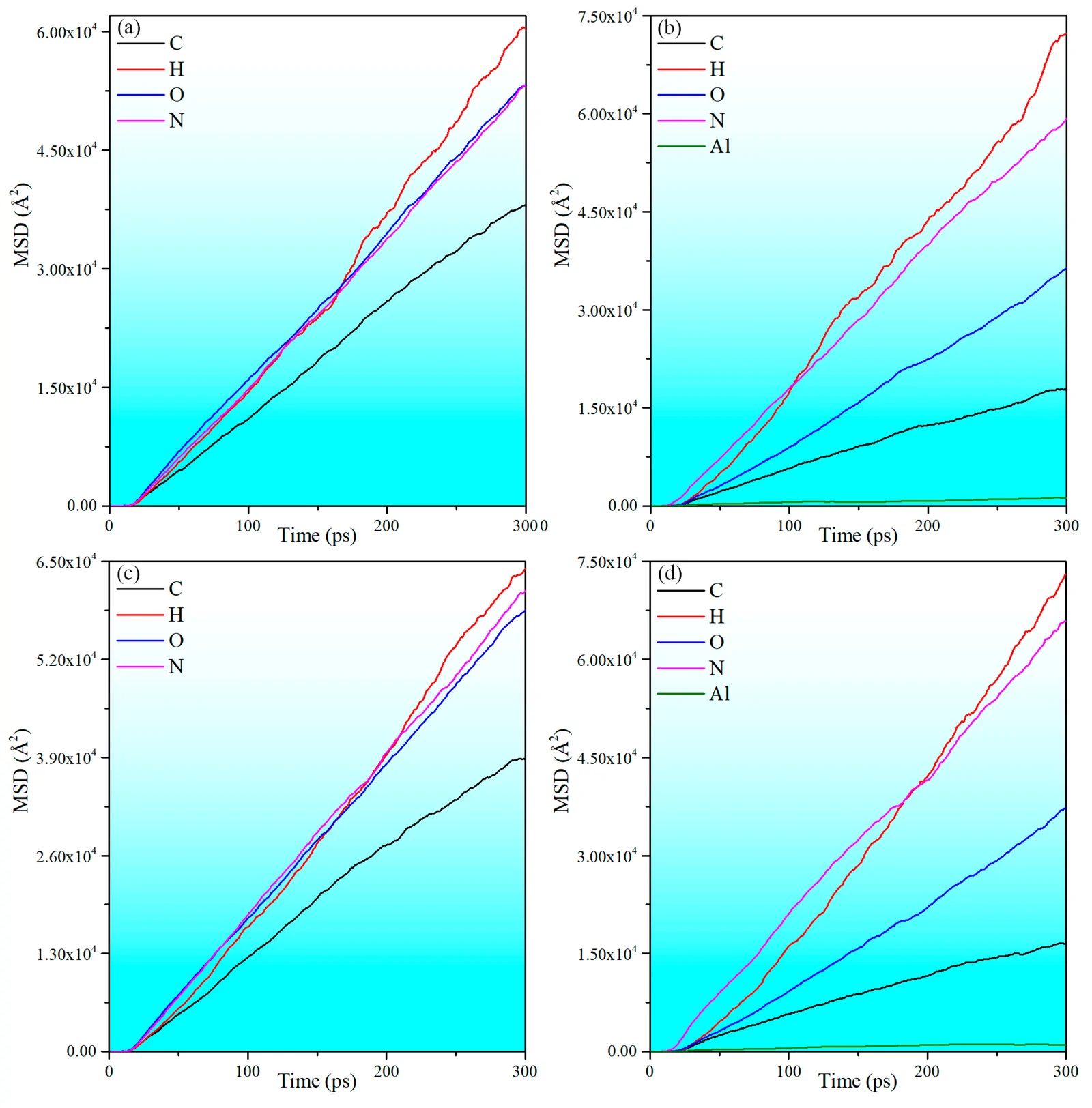
MSD curves of atoms in different systems: CL-20/BTF (**a**), CL-20/BTF@Al (**b**), CL-20/TNT (**c**), and CL-20/TNT@Al (**d**).

To fully characterize the dynamic evolution and atomic diffusion behaviors of the reaction system, the diffusion properties of atoms were investigated. The diffusion coefficient (D) could be derived from the correlation between the diffusion coefficient and mean square displacement (MSD), described by Einstein’s equation [30]. This work selected MSD curves within the range of 0–300 ps for least squares linear fitting to obtain the diffusion coefficients, and MSD statistical data was output by LAMMPS software. MSD curves can reflect the atomic self-diffusion characteristics, and Figure 6 presents the MSD curves of C, H, O, N, and Al atoms. The MSD curves for all atoms rose as time progressed, as demonstrated in Figure 6. At the early reaction stage, atomic MSD values remained relatively low. This phenomenon originated from the insufficient decomposition of energetic molecules, which imposed constraints on atomic motion. As the reaction proceeded, molecules continuously decomposed into atoms and radicals, thereby freeing atomic movement. Table 1 lists the atomic diffusion coefficients for different systems, while Table 2 shows the R^2^ and the standard error of the linear fitting of MSD curves, indicating that the fitting results are reasonable. For the thermal decomposition of CL-20/BTF and CL-20/TNT NPs, H atoms possess the maximum diffusion coefficient, whereas C atoms exhibit the minimum value. This could be explained by the minimal mass of H atoms, which yielded the highest thermal-motion velocity under identical thermal energy conditions. O atoms mostly existed in the form of small molecules and free radicals, followed by diffusion ability, and N atoms partially participated in the formation of a rigid heterocyclic skeleton; therefore, its diffusion was relatively weak. The C atom was the main body of the molecular skeleton, which easily polymerized to form multi-ring fragments and carbon clusters, with the lowest diffusion. Compared with the pure cocrystal systems, the diffusion of H and N atoms was increased in the Al-coated cocrystal systems, while the diffusion of C and O atoms showed a significant decrease. This was due to the release of a large amount of heat during the Al reaction, which promoted the decomposition of intermediates and increased the number of free H- and N-containing small molecules in the system, resulting in an increase in the diffusion of H and N. Al easily combined with O to form stable clusters, while the cluster interface adsorbed bound carbon fragments, greatly limiting the migration of C and O, resulting in a significant decrease in their diffusion.

## 3. Conclusions

This study used RMD simulations to systematically investigate the thermal decomposition characteristics of Al-coated CL-20-based cocrystal explosive (CL-20/BTF@Al and CL-20/TNT@Al) NPs at 2500 K, and to reveal how metallic Al exerts regulatory effects on the thermal decomposition mechanism of energetic materials by comparison with the pure CL-20-based cocrystal system. The results indicate that Al not only generates abundant local high-temperature hotspots that serve as active sites for initiating chemical reactions; it also retards heat loss and promotes temperature rise. The evolution of thermal decomposition products differs markedly between Al-coated and pure cocrystal systems, confirming that Al effectively regulates the decomposition pathway. Atomic diffusion analysis shows that heat released by Al reactions accelerates intermediate decomposition, increasing the concentrations of free H- and N-containing small molecules and thereby enhancing the diffusion coefficients of H and N atoms. Meanwhile, Al readily combines with O to form stable clusters whose interfaces adsorb carbon fragments, significantly suppressing C and O migration and reducing their diffusion coefficients.

Although this study has revealed several new insights, it still has certain limitations. The nanoscale system size and the 300 ps time scale cannot fully reproduce the microstructural complexity and slow aging processes of real Al-coated CL-20-based cocrystal explosives. Moreover, the high simulation temperatures promote multi-channel reactions and shorten intermediate lifetimes, yielding high-temperature-limit decomposition behavior. Consequently, the simulated decomposition pathways, product distributions, and hotspot characteristics should be interpreted as idealized interfacial results, and quantitative extrapolation to low-temperature experimental conditions should be avoided.

This work clarifies the microscopic thermal reaction mechanism of metal/energetic composites from an atomic-scale perspective, which provides an essential theoretical foundation for component formulation and performance optimization of high-efficiency energetic composites. Future studies should extend the present ReaxFF-MD framework toward more experimentally relevant conditions. This includes increasing system size, extending the simulation time scale through accelerated sampling or kinetic modeling, and performing simulations at lower heating rates. Coupling these simulations with experimental thermal analysis would help validate decomposition pathways, product distributions, and hotspot behavior, and support the development of multiscale models for assessing the thermal stability and safety of Al-coated CL-20-based cocrystal explosives.

## 4. Computational Methods

**Figure 7:**
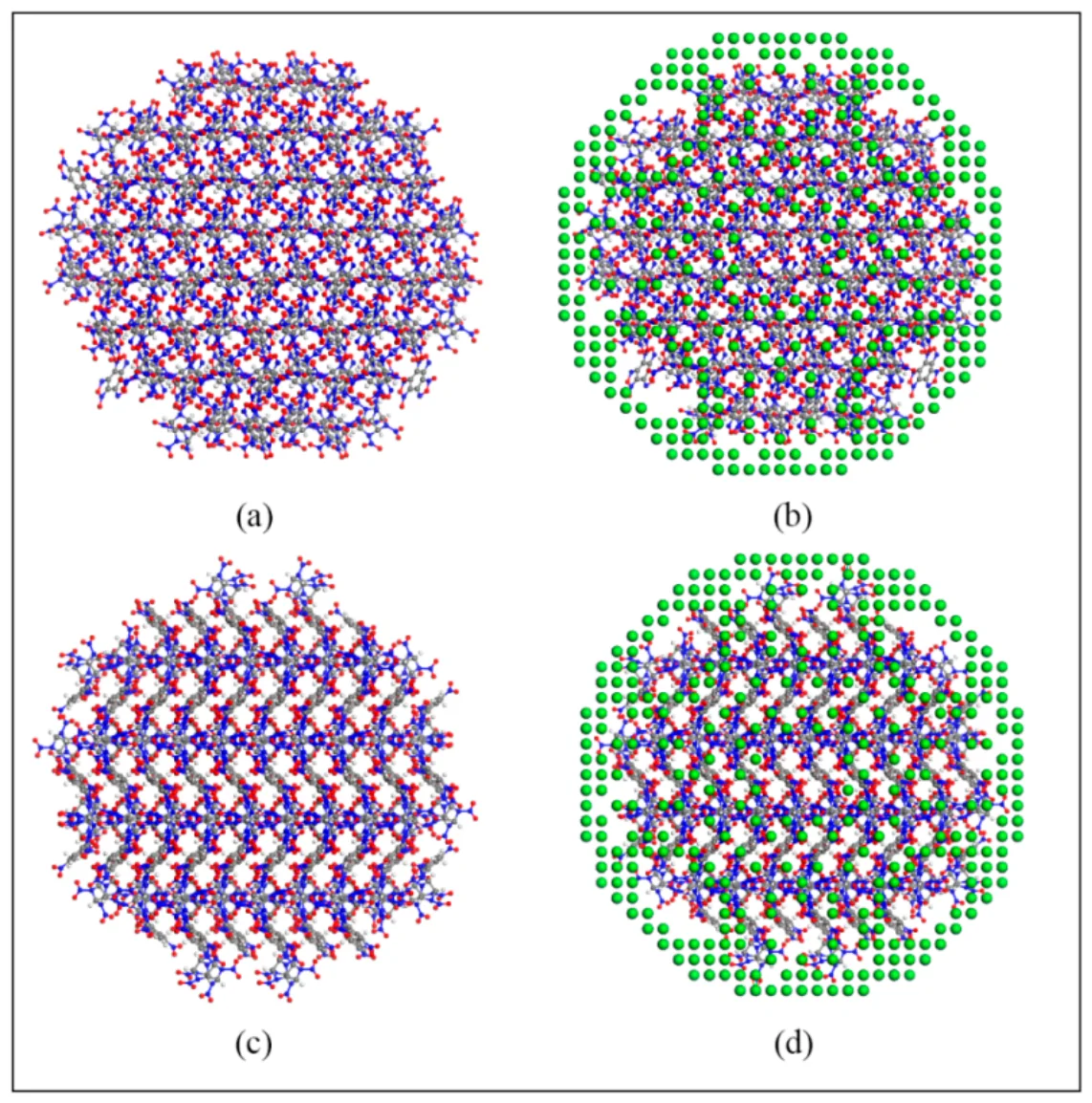
Schematic diagram of nanoparticles: CL-20/BTF (**a**), CL-20/BTF@Al (**b**), CL-20/TNT (**c**), and CL-20/TNT@Al (**d**). The C, H, O, N and Al are represented by gray, white, red, blue and green balls, respectively.

The unit cells of CL-20/BTF and CL-20/TNT were obtained from experimental data [7,8]. First, CL-20/BTF (containing 111 CL-20 and 112 BTF molecules) and CL-20/TNT (containing 112 CL-20 and 116 TNT molecules) NPs with a diameter of 5 nm were constructed. Then, the Al shell with a diameter of 6 nm and a thickness of 0.1 nm was constructed, which contained 720 Al atoms. The Al shell adopted concentric spherical geometry: a 6 nm outer-diameter Al sphere was constructed, from which a concentric inner sphere of 5.8 nm diameter was subtracted. The residual Al atoms confined in the spherical annulus form a shell with a geometric thickness of 0.1 nm. This value was geometrically defined and did not represent a close-packed continuous atomic monolayer. Subsequently, CL-20/BTF@Al and CL-20/TNT@Al NPs with core–shell structures were constructed, and the mass fractions of Al in the system were 20.2% and 20.5%, respectively. The structure of these NPs is shown in Figure 7.

**Figure 8:**
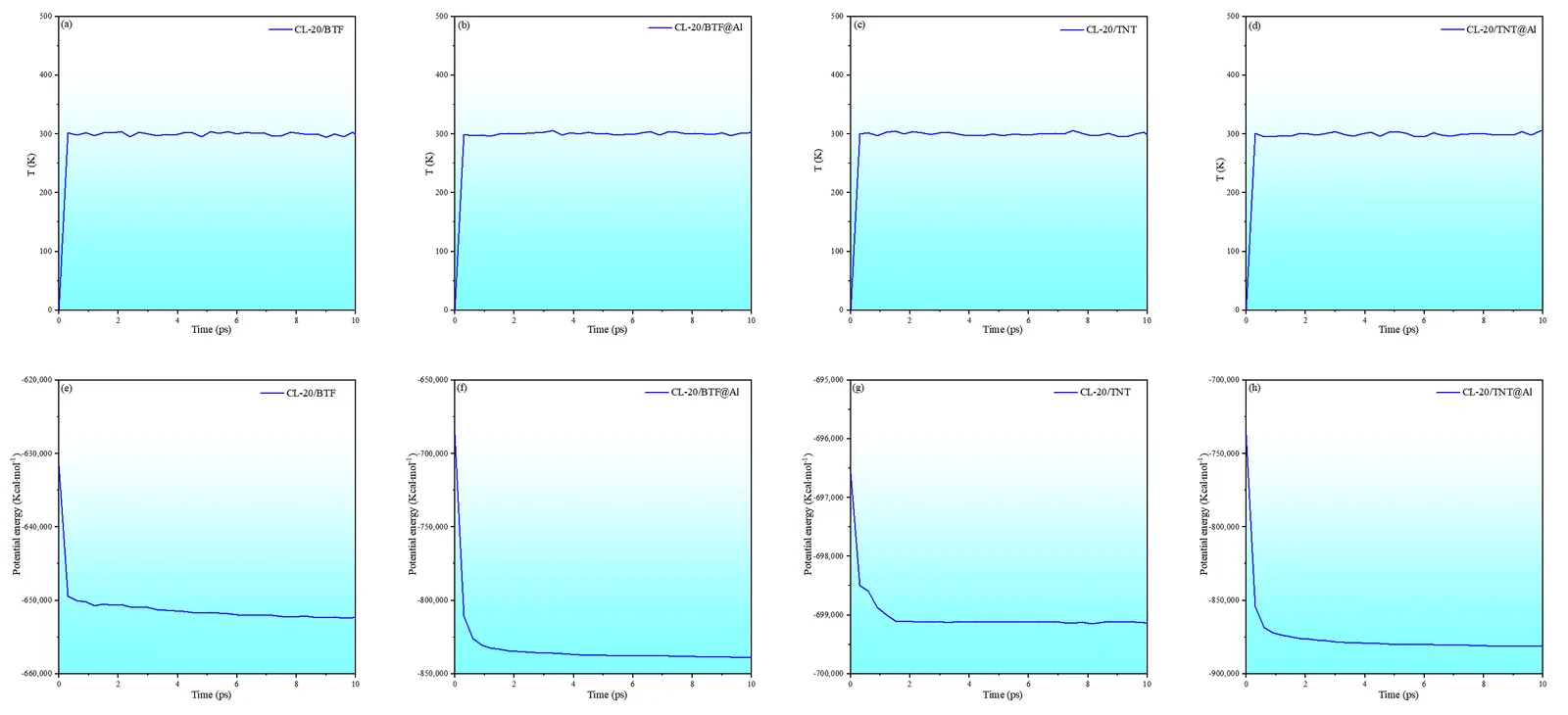
The evolution curves of temperature (**a**–**d**) and potential energy (**e**–**h**) for different systems during the relaxation process.

All the simulations were carried out using the ReaxFF implemented in LAMMPS [31], and the ReaxFF parameter-set used in this work was developed for C/H/O/N/Al by Mei et al. [32]. The Ovito package [33] was used for visualizing product structures, and the bond order cutoff was set to 0.3 to avoid errors caused by an excessively small cutoff that could result in numerous bonds or an excessively large cutoff that could trigger molecular fragmentation [34]. The automatic reaction network generator (ReacNetGenerator) for RMD simulations was used to analyze the number of fragments [35]. These four NPs were placed in cubic boxes with a side length of 8 nm as the initial model of thermal decomposition before the RMD simulation. The explosives would produce extremely high temperatures during the detonation process, and the detonation temperature could be above 2000 K. The total simulation time and calculation efficiency were considered; thus, 2500 K was selected as the simulation temperature [36]. The system was equilibrated for 10 ps via the NPT ensemble at 300 K, and Figure 8 presents the evolution curves of temperature and potential energy for different systems during the relaxation process. It can be seen that the temperature and potential energy and volume of all systems became stable without obvious fluctuations after 10 ps of relaxation, indicating that the systems reached an equilibrium state after 10 ps of relaxation. Subsequently, the equilibrated system was heated to 2500 K within 25 ps under the NVT ensemble to simulate thermal decomposition. The total simulation time was 300 ps with a time-step of 0.1 fs, and the velocity-Verlet algorithm was used to solve Newton’s equations of motion. The Nosé–Hoover thermostat was used for NVT temperature control, with a thermal bath time constant of 50 fs and an additional drag coefficient of 0.2 to suppress intrinsic resonance oscillations.

## Figures and Tables

**Table 1:** Diffusion coefficients of C, H, O, N, and Al atoms in different systems (D/×10^−8^ m^2^.s^−1^).

	C	H	O	N	Al
**CL-20/BTF**	24.98	38.94	33.72	33.71	
**CL-20/BTF@Al**	10.60	41.80	21.31	35.05	0.61
**CL-20/TNT**	23.54	38.51	34.10	35.48	
**CL-20/TNT@Al**	9.87	43.19	21.63	37.66	0.65

**Table 2:** R^2^ and standard error of linear fitting of MSD curves.

	C		H		O		N		Al	
	R^2^	Standard Error	R^2^	Standard Error	R^2^	Standard Error	R^2^	Standard Error	R^2^	Standard Error
**CL-20/BTF**	0.998	0.352	0.996	1.258	0.999	0.341	0.998	0.434		
**CL-20/BTF@Al**	0.997	0.204	0.993	1.153	0.996	0.442	0.998	0.413	0.964	0.047
**CL-20/TNT**	0.997	0.449	0.994	0.999	0.999	0.247	0.999	0.380		
**CL-20/TNT@Al**	0.995	0.231	0.991	1.478	0.995	0.498	0.998	0.446	0.943	0.069

## Data Availability

The datasets generated and analyzed during the current study are available from the corresponding author upon reasonable request.
